# Comparison of the effects of left atrial appendage closure and oral anticoagulants in preventing stroke in patients with non-valvular atrial fibrillation: A protocol for systematic review and meta-analysis

**DOI:** 10.1097/MD.0000000000027251

**Published:** 2021-09-17

**Authors:** Deyong Yue, Yunda Jiang, Zhongying Yang, Liang Cao, Long Huo, Jing Wang

**Affiliations:** a Department of Pharmacy, Chongming Branch of Xinhua Hospital Affiliated to Shanghai Jiao Tong University School of Medicine, No. 25 Nanmen Road, Chengqiao Town, Chongming District, Shanghai, China; b Department of Information, Chongming Branch of Xinhua Hospital Affiliated to Shanghai Jiao Tong University School of Medicine, No. 25 Nanmen Road, Chengqiao Town, Chongming District, Shanghai, China; c Department of Spleen and Stomach Diseases, Longhua Hospital Affiliated to Shanghai University of Traditional Chinese Medicine, No. 725 South Wanping Road, Xuhui District, Shanghai, China; d Department of Internal Medicine of Traditional Chinese Medicine, Chongming Branch of Xinhua Hospital Affiliated to Shanghai Jiao Tong University School of Medicine, No. 25 Nanmen Road, Chengqiao Town, Chongming District, Shanghai, China.

**Keywords:** left atrial appendage closure, meta-analysis, non-valvular atrial fibrillation, oral anticoagulants, stroke

## Abstract

**Background:**

This study aims to analyze and evaluate the difference in efficacy between left atrial appendage closure (LAAC) and oral anticoagulants (OA) in preventing stroke in patients with non-valvular atrial fibrillation (NVAF) through the method of meta-analysis. The purpose is to provide for the prevention of stroke in patients with NVAF valuable treatment guidance.

**Methods:**

This study is a comprehensive collection of randomized controlled studies of LAAC and OA in the prevention of stroke in patients with NVAF, and searches PubMed, Embase, the Cochrane Library, Web of Science, CNKI, SinoMed, VIP Database, WANFANG Database, and other Chinese and English databases by combining subject words with free words, and the retrieval time is from the establishment of each database to June 1, 2021. At the same time, searching the included literature and literature of related reviews by manual. Two researchers independently conduct literature screening and quality evaluation. Statistical software RevMan 5.3 and Stata 12.0 were used for meta-analysis.

**Results:**

This study evaluating the difference in efficacy between LAAC and OA in preventing stroke in patients with NVAF will be published in high-quality medical academic journals.

**Conclusion:**

This study will give the best treatment strategy to prevent stroke in patients with NVAF, and provide some reference for clinical medical staff.

OSF registration number: DOI 10.17605/OSF.IO/2UXPA (https://osf.io/2uxpa).

## 1 Introduction

Atrial fibrillation (AF) is the most common arrhythmia symptom in clinic. 3% to 5% of people over 65 years old are affected by atrial fibrillation, and their prevalence and incidence rate increase with age.^[1,2]^ Due to the changes of electrical activity, mechanical activity, nerve conduction, and other mechanisms in patients with atrial fibrillation, the heart produces ineffective contraction and the decline of ejection ability. At the same time, atrial fibrillation can change hemodynamics. Abnormal hemodynamics makes it easy to produce thrombus or plaque shedding, which leads to systemic embolism. Embolic stroke is one of its serious complications.^[3–5]^ The formation of thrombus is the link that causes non-valvular atrial fibrillation (NVAF) to become a major risk cause of stroke. According to reports, about 90% of the thrombus in NVAF patients is formed in the left atrial appendage, but the etiology of thrombosis in the left atrial appendage is not clear.^[6,7]^ Therefore, the focus of stroke prevention in NVAF patients is how to prevent thrombosis, which is what we often call anticoagulation therapy. Although oral anticoagulants (OA) are currently the standard plan to prevent stroke in NVAF patients, due to various reasons such as bleeding risk, compliance, and cost, especially elderly patients with atrial fibrillation have poor compliance with anticoagulants. However, 50% of patients are still considered unsuitable for OA.^[8,9]^ For patients with atrial fibrillation who are at high risk of bleeding or cannot tolerate OA, left atrial appendage closure (LAAC) is an alternative therapy to reduce the risk of thromboembolic events. The 2019 AHA Guidelines for the Management of Patients with Atrial Fibrillation and the 2020 ESC Guidelines for the Diagnosis and Management of Atrial Fibrillation clearly point out that LAAC is particularly suitable for patients with NVAF who are at high risk of stroke and have contraindications to long-term anticoagulation therapy.^[10,11]^

Although there are increasing clinical studies on the prevention of stroke in NVAF patients by LAAC, there is a lack of evidence-based medical evidence. The purpose of this study is to analyze and evaluate the efficacy of LAAC and OA in the prevention of stroke in patients with NVAF through systematic evaluation and meta-analysis, so as to provide guidance for selecting appropriate methods to prevent stroke in patients with NVAF.

## 2 Methods

### 2.1 Protocol registration

OSF registration number: DOI 10.17605/OSF.IO/2UXPA (https://osf.io/2uxpa). August 17, 2021.

### 2.2 Inclusion and exclusion criteria

#### 2.2.1 Types of studies

A comprehensive collection of randomized controlled studies on the clinical efficacy of LAAC and OA in preventing stroke in patients with NVAF. The publication language of the literature is set to Chinese or English.

#### 2.2.2 Types of participants

Patients with NVAF confirmed by clinical diagnosis.

#### 2.2.3 Inclusion criteria

(1) The subjects were patients with atrial fibrillation indicated by electrocardiogram.
(2) The experimental group was patients with LAAC, and the control group was patients with oral anticoagulants.
(3) There are detailed outcome indicators. The outcome indicators include at least the following 3 items: ischemic stroke, hemorrhagic stroke, peripheral vascular embolism events, total bleeding events, cardiovascular events, etc.

#### 2.2.4 Exclusion criteria

(1) Does not meet the inclusion criteria.
(2) There are no above outcome indicators, insufficient original data, or no full-text literature.
(3) The literature type collected does not meet the requirements non-randomized controlled studies, reviews, case reports, animal experiments, etc.

#### 2.2.5 Interventions

Control group: OA were used alone to prevent stroke in patients with NVAF. Experimental group: LAAC was used alone to prevent stroke in patients with NVAF. All patients were given the necessary basic treatment.

### 2.3 Types of outcome measures

The main outcome indicators: ischemic stroke, peripheral vascular embolism events, cardiovascular events. The secondary outcome indicators: hemorrhagic stroke, massive hemorrhage, all-cause death, and sudden cardiac death.

### 2.4 Search strategy

Using the computer to search PubMed, Embase, the Cochrane library, Web of Science, CNKI, SinoMed, VIP Database, WANFANG Database, screening the related literature from the establishment of databases to June 1, 2021. After the literature retrieval completes, carefully reading the relevant review or similar references cited by meta-analysis research institute, searching the references to avoid search omissions. The retrieval method is developed by combining mesh subject words with free words and Boolean logic operators, the subject words and free words are queried through the PubMed database.

### 2.5 Data collection and analysis

**Figure 1:**
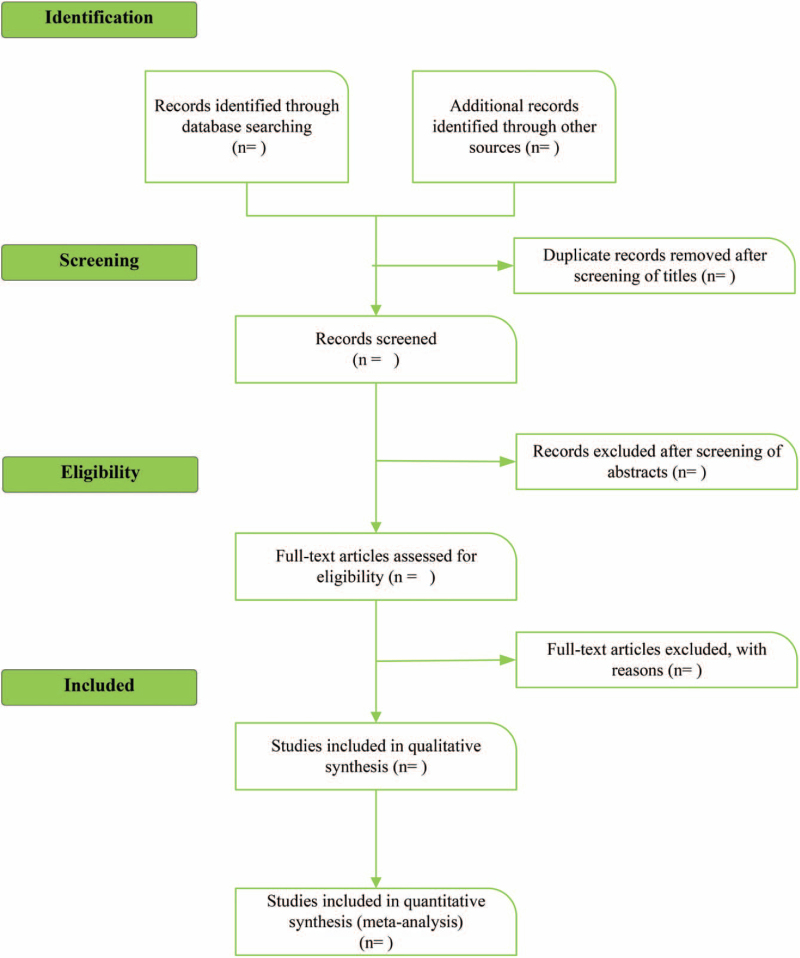
Flow chart of literature screening.

Two researchers strictly follow the inclusion and exclusion criteria, search independently according to the designed retrieval strategy, browse the title and abstract, select relevant literature and exclude obviously unrelated literature, read the full text of the literature that may meet the inclusion criteria, determine whether to include the study and crosscheck. In case of disagreement, both parties should discuss or consult the third researcher. Data extraction included: basic information of literature, intervention measures and intervention time, and outcome indicators. The flow chart of literature screening is shown in Figure 1.

### 2.6 Risk of bias assessment

Conducting the bias risk evaluation to the selected literature based on the method recommended by Coachrane Reviewe's Handbook 5.1.0 and its results are reported.^[17]^ The quality assessment is independently assessed by the 2 researchers, and if consensus could not be reached, it is discussed with the third researcher. Each of the 7 indicators is classified into 3 levels (i.e., low-level bias risk, unclear bias risk, high-level bias risk). Finally, the evaluation results are input into Revman 5.3 software to draw a summary chart of bias risk.

### 2.7 Statistical analysis

#### 2.7.1 Data synthesis

Using RevMan 5.3 software to conduct meta-analysis on the collected data. The second classification variable uses odds ratios and 95% CI for data analysis, and the continuous variable uses mean difference and 95% confidence interval for data analysis. Heterogeneity is evaluated using *χ*^2^ and *I*^2^, when *I*^2^ ≤ 50% heterogeneity is small, through a fixed effect model to analyze the extracted data, when *I*^2^ > 50% heterogeneity is greater, through a random effect model to analyze the extracted data. Subgroup analysis or sensitivity analysis can be performed when the heterogeneity of the study is obvious.

#### 2.7.2 Sensitivity analysis

After excluding low-quality studies, the combined effect size is re-estimated. If the results after the exclusion do not change significantly compared with those before the exclusion, the sensitivity is low and the results are more robust and credible; conversely, if the conclusions after exclusion are large or even diametrically opposed, the sensitivity is higher, the robustness of the results is low, and caution must be taken in interpreting the results and reaching conclusions.

#### 2.7.3 Subgroup analysis

This study will use subgroup analysis of indicators with high heterogeneity, selecting factors that are highly correlated with the outcome indicators of this study as the grouping basis for subgroup analysis, and further evaluating the reliability of the results obtained.^[12]^

#### 2.7.4 Publication bias

Funnel plots are often used to test the publication bias of each study, if the presented funnel plot is asymmetrical to the left and right, or if there is a missing angle, it is suggested that the included study may have publication bias. It is generally accepted that funnel plot is required when the inclusion literature is more than 8 articles.^[13]^

### 2.8 Ethics and dissemination

This research is a secondary study of published documents, and does not involve humans or animals. The data used are obtained from published papers. Based on this, this study does not involve ethical issues.

## 3 Discussion

Atrial fibrillation and stroke are major health problems in today's society. They have common risk factors and often coexist. Among them, ischemic stroke is one of the complications worthy of attention in patients with atrial fibrillation.^[14]^ The main type of atrial fibrillation is NVAF, and its clinical burden is mainly the increased risk of thromboembolic events. Compared with patients without NVAF, thromboembolism in NVAF patients leads to a greater risk of stroke recurrence, worse functional status, and mortality Higher.^[15]^ Therefore, the prevention of stroke through appropriate antithrombotic therapy is the core of NVAF treatment. Numerous studies have also confirmed that standard anticoagulation therapy can significantly reduce the risk of embolism in NVAF patients. What still needs to be considered when using anticoagulant drugs is the increased risk of bleeding, especially major bleeding that may require hospitalization, blood transfusion, and surgical intervention, or major bleeding involving important anatomical locations, such as intracranial hemorrhage, which is significantly related to increased mortality.^[16]^ Therefore, not all NVAF patients are suitable targets for anticoagulation, and some NVAF patients are only suitable for short-term anticoagulation. Therefore, the study of non-pharmacological treatment of NVAF patients is also crucial, because the thrombosis of NVAF patients is most common in the left atrial appendage, so LAAO has become a potential non-pharmacological treatment for stroke prevention in NVAF patients.^[17]^ However, there is currently a lack of evidence-based medical evidence comparing the efficacy of LAAO and OAC in preventing stroke in patients with NVAF. The curative effect that occurs is compared and evaluated, and a definite conclusion will be given through this study, which will benefit more NVAF patients.

## Author contributions

**Conceptualization:** Jing Wang, Deyong Yue.

**Data curation:** Deyong Yue, Yunda Jiang, Zhongying Yang, Liang Cao, Long Huo.

**Funding acquisition:** Jing Wang.

**Methodology:** Deyong Yue, Yunda Jiang.

**Resources:** Deyong Yue, Zhongying Yang, Liang Cao.

**Software:** Yunda Jiang, Zhongying Yang, Long Huo.

**Writing – original draft:** Deyong Yue, Yunda Jiang.

**Writing – review & editing:** Jing Wang.
